# The Photocatalytic Activity of Photoresponsive Silver Nanoparticle/Zinc Oxide Composite Thin Films with Unprecedently Elevated Quantities of Silver

**DOI:** 10.3390/nano16060340

**Published:** 2026-03-10

**Authors:** Likius Shipwiisho Daniel, Patemasella Gawanas, Alina Uusiku, Willem Pendukeni Nashidengo, Ateeq Rahman, Kassian T. T. Amesho, Veikko Uahengo

**Affiliations:** 1 Science and Technology Division, Multidisciplinary Research Service, Centre for Research Service, University of Namibia, Private Bag 13301, 340 Mandume Ndemufayo Avenue, Windhoek 10005, Namibia; 2 Department of Physics, Chemistry and Material Science, School of Science, Faculty of Agriculture, Natural Science and Engineering, University of Namibia, Private Bag 13301, 340 Mandume Ndemufayo Avenue, Windhoek 10005, Namibia; arahman@unam.na (A.R.); vuahengo@unam.na (V.U.); 3 Analytical Chemistry Lab, Department of Infrastructure Water Technical Services, Windhoek 10005, Namibia; 4 Namibia Green Hydrogen Research Institute, University of Namibia, Private Bag 13301, 340 Mandume Ndemufayo Avenue, Windhoek 10005, Namibia; auusiku@unam.na; 5 Department of Applied Educational Science, School of Education, P.O. Box 59, Oshakati 5530, Namibia; wnashidengo@unam.na; 6 Department of Higher Education and Lifelong Learning, School of Education, Faculty of Education and Human Sciences, University of Namibia, Private Bag 13301, 340 Mandume Ndemufayo Avenue, Windhoek 10005, Namibia; kamesho@unam.na

**Keywords:** photocatalytic efficacy, silver nanoparticles (Ag-NPs), zinc oxide (ZnO) thin films, surface plasmon resonance (SPR), molecular precursor method (MPM), visible light responsiveness

## Abstract

The photocatalytic efficacy of metallic silver nanoparticle/zinc oxide (Ag-NPs/ZnO) composite thin films, COMP-Ag_x_, with varying silver concentrations (0 mol% ≤ x ≤ 100 mol%), is investigated for the degradation of methyl orange (MO). The films were spin-coated on a silica glass surface at 600 °C utilizing the molecular precursor method (MPM). The XRD spectra of these composite thin films revealed three significant peaks corresponding to the diffraction planes of (0 0 2), (1 0 0), and (1 0 1), indicative of the formation of ZnO crystallites in diverse orientations, in conjunction with an additional signal for cubic Ag crystals. The magnitude of the ZnO peaks diminishes as the mol% of silver increases. The images from the SEM confirm the integration of Ag-NPs into the ZnO matrix. The UV/Vis absorption spectra exhibit a 410 nm surface plasmon resonance (SPR) peak for composite Ag-NP/ZnO thin films. The absorption spectra of ZnO and Ag-NP/ZnO composite thin films demonstrate the band gap of ZnO to be 3.4 eV, while the band gaps of the composite thin films nearly approximate that of ZnO. The decomposition rates of the MO solution indicate that composite thin films function effectively under visible irradiation compared to pure ZnO. The optical properties indicated that the SPR of Ag-NPs contributed to the visible responsiveness of the composite thin films. The SPR demonstrate significant visible light responsiveness and essential characteristics during photoexcited electron transfer from the Ag-NPs to the ZnO conduction band.

## 1. Introduction

Zinc oxide (ZnO) is a multidimensional semiconductor with a broad direct band gap of approximately 3.37 eV and a large exciton binding energy of 60 meV at room temperature [1,2]. These properties brand ZnO as the ultimate candidate for a variety of optoelectronic, photocatalytic, and sensing applications. ZnO thin films have garnered considerable interest owing to their superior optical transparency, chemical stability, and straightforward fabrication via diverse deposit techniques, such as sol–gel [3], sputtering [4], pulsed laser deposition [5], and molecular precursor methods (MPMs) [6]. In optoelectronics, ZnO thin films are essential parts of ultraviolet (UV) photodetectors [7], light-emitting diodes (LEDs) [8], solar cells [9], and transparent conducting electrodes [10]. Their capacity to effectively absorb UV radiation renders them appropriate for display technologies and UV-blocking applications. Moreover, the piezoelectric characteristics of ZnO render it advantageous for acoustic wave devices and flexible electronics [11].

ZnO thin films are intensively researched for their photocatalytic characteristics in restoration of the environment [12,13]. When exposed to UV radiation, ZnO generates electron–hole pairs, initiating redox reactions that degrade organic contaminants and dyes in wastewater treatment [14]. Nonetheless, despite its advantageous properties, pure ZnO encounters specific restrictions that impede its efficacy in optical and photocatalytic applications. ZnO experiences a recombination of photogenerated electron–hole pairs, diminishing its photocatalytic efficacy [15]. This constrains its capacity to maintain extended redox reactions. ZnO is confined to the absorption of visible light. ZnO’s high bandgap limits its absorption to the ultraviolet area, which accounts for just about 5% of the solar spectrum. This constraint decreases its efficiency under natural sunlight. Furthermore, extended UV exposure can cause self-degradation, which reduces the long-term stability and reusability of ZnO-based photocatalysts [16]. Many strategies have been investigated to overcome these restrictions, including doping, composite synthesis, and nanostructuring [17,18]. Silver (Ag) doping has been shown to effectively improve the optical and photocatalytic characteristics of ZnO [19,20]. Doping ZnO thin films with silver (Ag) increases their optical and photocatalytic properties. The incorporation of Ag nanoparticles (Ag-NPs) into ZnO modifies its electrical properties, causes surface plasmon resonance (SPR), and enhances charge carrier dynamics [20]. Ag-NPs demonstrate localized surface plasmon resonance (LSPR) effects, resulting in enhanced absorption within the visible light spectrum and prolonging the photocatalytic efficacy of ZnO beyond the ultraviolet range [15]. Ag functions as an electron trap, inhibiting the fast recombination of photogenerated charge carriers, thus enhancing photocatalytic effectiveness [21]. The use of Ag modifies the band structure of ZnO, reducing the bandgap and enhancing visible light absorption [5,17].

The Ag loading in ZnO has been examined for its photocatalytic characteristics. The photocatalytic activity of Ag-doped nanoparticles (Ag-NPs) was reported to be superior under direct sun irradiation. El-Bindary and colleagues [22] investigated the photocatalysis of reactive blue 21 by utilizing Ag-NPs/ZnO nanoparticles synthesized via the co-precipitation technique with Ag concentrations ranging from 1% to 7%. The improvement in photocatalytic activity resulted from the modification of ZnO with a suitable quantity of Ag to boost the separation efficient splitting of photogenerated electrons and holes in ZnO. Diantariani et al. [23] investigated the photodegradation of methylene blue with an Ag-NPs/ZnO photocatalyst. The Ag loadings (1.7–4.4%) of ZnO demonstrated increased photocatalytic activity relative to undoped ZnO. Ahmad and Jaffri [24] recorded photosynthetic Ag-doped ZnO nanoparticles at 0.2–2% doping for the photodegradation of safranin O, rhodamine B, and methyl orange. The researchers contend that the most heavily doped Ag/ZnO may function as a locus for charge aggregation. The increased charge concurrence is linked to elevated Ag loading on ZnO, resulting in a strong attraction among negatively charged silver ions and the positively charged holes on the ZnO’s surface [25]. Moreover, elevated doping concentrations may adversely impact the UV photon consumption of ZnO, thus diminishing the overall photocatalytic efficacy of Ag-ZnO nanoparticles [26]. The composite thin film is theorized to facilitate the agglomeration of silver particles by employing a solution with a greater volumetric ratio of Ag-NPs, hence inhibiting a uniform dispersion of silver inside the dielectric metal oxide matrix. Li et al. [27] encountered challenges in obtaining a consistent solution via the sol–gel method when the silver content exceeded 18 mol%. The polymerization of metalloxanes in the media restricted their mixing capability [27].

This work evaluated the photocatalytic activity of zinc oxide integrated with an elevated doping concentrations of Ag-NPs up to 80% within the ZnO matrix, synthesized via the molecular precursor method (MPM). The MPM approach, utilizing chemical techniques, was first studied in 1996 [28,29]. This technology is a potential one-step method that ensures superior uniformity and precise control over the concentration and distribution of dopants inside the metal oxide matrix [30]. Through the utilization of the MPM approach, the production of ZnO composite thin films has been successfully obtained [6]. In this paper, the MPM approach was employed in the design of the Ag precursor to generate Ag-NPs/ZnO composite thin films with elevated silver concentrations.

This study demonstrates the ongoing incorporation of metallic Ag nanoparticles within the ZnO, displaying surface plasmon resonance (SPR) characteristics within the visible light spectrum [5,17,20]. The electron transport mechanism facilitated by surface plasmon resonance in a noble metal/ZnO composite system at elevated silver concentrations remains inadequately elucidated. Hence, we evaluate the photodegradation of methyl orange (MO) dye using Ag-NPs/ZnO composite thin films, featuring an exceptionally high incorporation of metallic silver nanoparticles within ZnO by using an MPM approach, i.e., a chemical approach. The present investigation demonstrates that the MPM, which provides superior miscibility of the silver salts and Zn-EDTA precursor solutions, effectively addresses the miscibility limitations of the conventional sol–gel method and is essential for producing composite thin films with a high molar percentage (mol%) of silver [27]. It further illustrates that surface plasmon resonance (SPR) induces electron transfer from silver nanoparticles (Ag-NPs) to zinc oxide (ZnO), resulting in charge separation initiated by visible light [21]. This contradicts what is commonly documented in the literature [20]. The metallic silver, crucial for the recombination of electron–hole pairs on the surface of ZnO, swiftly converts into silver ions when subjected to water solutions in aerobic conditions. Nonetheless, this does not apply to the composite thin films of silver nanoparticles (Ag-NP) and ZnO fabricated via the MPM method. The Ag-NPs/ZnO composite thin films, containing up to 70 mol% Ag in the ZnO matrix, exhibit photocatalytic activity when subjected to visible light irradiation.

## 2. Materials and Methods

### 2.1. Materials

Zinc acetate dihydrate, ethylenediaminetetraacetic acid (EDTA), 2-methoxyethanol, dibutyl amine, monoethanolamide (MEA), pure C_2_H_5_OH, and pure CH_3_OH were procured from Merck, CT, South Africa. All reagents were utilized without any additional processing.

The following chemicals were procured from J.J Nam Chemicals (Pty) Ltd., situated in Windhoek (WDH), Namibia: silver acetate, ethanol, hydrogen peroxide (30% purity), and ethylenediamine-N,N,N’,N’-tetra acetic acid (EDTA). The chemicals utilized in the investigation were employed without further purifying procedures. Silica glass slides (1.5 × 10 × 10 mm^3^) were used as substrates for the coating of ZnO thin films. Silica glass specimens of 100 × 100 × 1.5 mm^3^ were obtained from Burbridge Glass CC. WDH, Namibia The glass substrates were prepped and washed with propan-2-ol in a sonicator. This method sought to eradicate physiosorbed organic molecules potentially present on the surfaces. Moreover, the procedure entailed several rinses with deionized water and subsequently with ethanol. The substrates were, thereafter, treated to a drying process in an oven maintained at a temperature of 70 °C.

### 2.2. Preparation of the Zn-EDTA Complex, Silver Acetate Precursor, and COMP-Ag-Zn Solutions by MPM

To formulate a precursor solution of the zinc–ethylenediaminetetraacetic acid (Zn-EDTA) complex, 7.24 g of EDTA was dissolved in 9.24 mL of dibutyl amine, followed by the addition of a mixture consisting of 12 ml of methanol and 12 ml of ethanol into the partially dissolved EDTA/dibutyl amine solution. To fully dissolve the solids, the solutions were poured into a flask and boiled under reflux for 30 min. The resulting solution was then cooled to ambient temperature, after which 4.44 g of zinc acetate dihydrate was meticulously introduced into the flask, followed by reflux heating for two and half hours. Following that, the precursor was permitted to cool, after which 0.53 g of hydrogen peroxide solution was incorporated. A concluding hot reflux of 30 min was performed, yielding a clear solution that acted as the starting point for the zinc–EDTA complex. On the other hand, a precursor solution, S_Ag_, consisting of silver acetate and ethanol, was formulated utilizing a previously revealed method, the MPM [31,32]. Silver acetate (0.24 g) and dibutylamine (0.56 g) were incorporated into 10 mL of ethanol. The solution was subjected to agitation using a magnetic stirrer while being stirred for 5 min.

### 2.3. Fabrication of ZnO, Ag-NPs, and Various Ag-NP/ZnO Composite Thin Films

The respective Zn-EDTA and S_Ag_ precursor solutions were employed to produce films of ZnO and Ag-NPs on silica glass plates measuring 10 × 10 × 1.5 mm^3^ via the spin-coating technique, executed in a two-phase process. The first phase was executed at a velocity of 500 revolutions per minute for a period of five seconds. The last phase involved operating at a velocity of 2000 rpm for 30 s, followed by heating to a temperature of 600 °C. To formulate the coating solutions for Ag-NP/ZnO composites, Zn-EDTA and S_Ag_ solutions with variable silver molar concentrations (x) were amalgamated to fabricate distinct composite precursor solutions (S_Ag_n/ZnO-EDTA). The concentrations (x) employed were 0, 10, 20, 30, 40, 50, 60, 70, and 80 mol% of S_Ag_. S_Ag_n/ZnO-EDTA solutions were also spin-coated on silica glass plates and heated to a temperature of 600 °C in the furnace. The resulting various Ag-NPs/ZnO composite thin films were denoted as COMP-Ag_n_, where, if n represents 10 mol% of Ag in ZnO, the sample was designated as COMP-Ag10, and this nomenclature was applied to all composite thin films. This led to the production of various unprecedentedly elevated quantities of silver in Ag-NP/ZnO composite thin films designated as COMP-Ag_n_, where *n* = 10, 20, 30, 40, 50, 60, 70, and 80, respectively.

### 2.4. Characterization of the of ZnO, Ag-NPs, and Various Ag-NP/ZnO Composite Thin Films

The silver particles, ZnO, and COMP-Agn films were characterized by X-ray diffraction (XRD) utilizing a Bruker AXS MXP-18 AHF22 X-ray diffractometer. The XRD analysis was performed using Cu-Kα radiation produced at 45 kV and 300 mA. A 0.3 incidence angle was employed using parallel beam lenses.

A field-emission scanning electron microscope (FE-SEM) (S-4200, Hitachi, Tokyo, Japan) was used for studying the surface morphology of the resulting films. The studies were conducted using an accelerating voltage of 5.0 kilovolts. The optical absorption spectra of the ZnO, Ag-NPs, and COMPAgn thin films were examined on silica glass substrates utilizing a UV/Vis spectrophotometer (U-2800, Hitachi). Measurements were performed within the 200–700 nm wavelength range utilizing the double-beam method. By applying the Tauc formula, the direct band gap, denoted by the symbol *E_g_,* of the ZnO was determined. The formula is shown in the following Equation (1):

(1) $$\alpha =\frac{A(E_{phot}-E_{g}{)}^{\frac{1}{n}}}{E_{phot}},$$

where *E_phot_ (hv)* represents photon energy, *A* is a constant, α is the absorption coefficient at a certain wavelength (nm), and n is set to 0.5, assuming values for the direct transition modality.

### 2.5. The Study of the Photocatalytic Activity of ZnO Thin Film and Various Ag-NP/ZnO Composite Thin Films

The analysis of methyl orange (MO) photodegradation in distilled water was performed utilizing the methodology developed in our laboratory [31]. Several different Ag-NP/ZnO composite thin films, each with a different molar percentage of Ag (mol%), were utilized as photocatalysts. Additionally, an unmodified ZnO thin film was also utilized. The photocatalysts were submerged in 2 × 10^−2^ mM (6.54 × 10^−3^ mg/L) of MO aqueous solutions overnight in the absence of light to attain adsorption equilibrium on the film surfaces. The samples were subsequently taken out, rinsed with distilled water, and desiccated. Each sample was then placed in a 1 × 10^−2^ mM(3.27 × 10^−3^ mg/L) MO concentration within a 10 mL solution. The photocatalytic efficiency of these photocatalysts was evaluated using two different types of light, namely visible light irradiation generated by a LAX-Cute lamp in the range of 400–700, and UV light irradiation in the range of 300–400 nm from Lax-UV. The light intensity was measured to be 5.9 mW/cm^2^, which is equivalent to 8.0 × 10^4^ lux. The identical procedure was conducted in darkness. The concentration of MO after t minutes, C(t), was determined using the following Equation (2), with the absorption peak value at 465 nm:

(2) $$C\left(t\right)=100\times \frac{abs(t)}{abs(0)}=µM,$$

where the absorption of the solution immediately before light irradiation and after t minutes of irradiation are denoted by the symbols Abs (0) and Abs (t), respectively. The degradation rate of MO was evaluated thrice for each film, and the initial degradation rate (k) values of the concentration after t minutes for a 1 × 10^−2^ mM (3.27 × 10^−3^ mg/L) MO aqueous solution were determined via photoreaction with each photocatalytic and a control at the intervals of 20 min for 3600 min. This was achieved by applying the least-squares approach to approximate a line for the function C(t) against t, utilizing data within the interval of 0 < t ≤ 360 min. The index of photocatalytic action rate (IPCA) of the film was computed utilizing the average value of k, applying the following Equation (3) as determined from reference [33]:

(3) $$\mathrm{IPCA}=10^{3}\;\times \;\frac{\left|kn\right|}{3}\;=\;\mathrm{nM}\;\min^{-1}$$

## 3. Results

### 3.1. Fundamental Characterization of Synthetic Thin Films

#### 3.1.1. Crystalline Structure and Particle Dimensions 

**Figure 1:**
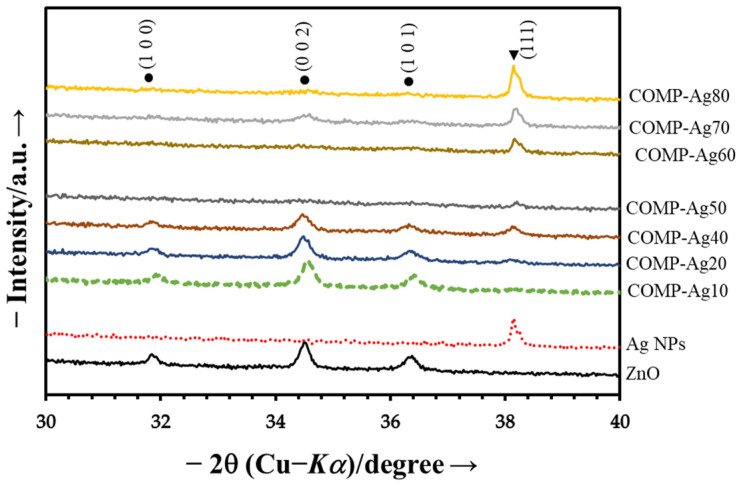
XRD patterns of the ZnO, Ag-NPs, and Ag/ZnO composite thin films. The peaks of each phase are represented as follows: ●: ZnO and ▼: silver.

Figure 1 illustrates the XRD patterns of ZnO, Ag-NPs, and the composite thin films deposited on silica glasses fabricated using the Zn-EDTA, S_Ag_ and COMP-Ag_n_/ZnO precursor solutions, respectively.

- The XRD spectra of the ZnO thin film reveal three prominent peaks associated with the diffraction planes of (1 0 0), (0 0 2), and (1 0 1), indicating the development of ZnO crystalline particles in various orientations.
- The peaks align with those of the standard ZnO [JCPDS 80-0075]. As a result, the conventional hexagonal wurtzite configuration of the thin films is deduced from the XRD signals [34].
- The peak at 38.5° in the XRD pattern of the silver film confirms the existence of metallic Ag-NPs [35].
- The intensity of this peak is shown to grow with the rising mol% of silver in the ZnO matrix, as demonstrated in the composite thin films.
- The intensity of ZnO peaks diminishes as the mol% of silver increases.

#### 3.1.2. Surface Morphology

**Figure 2:**
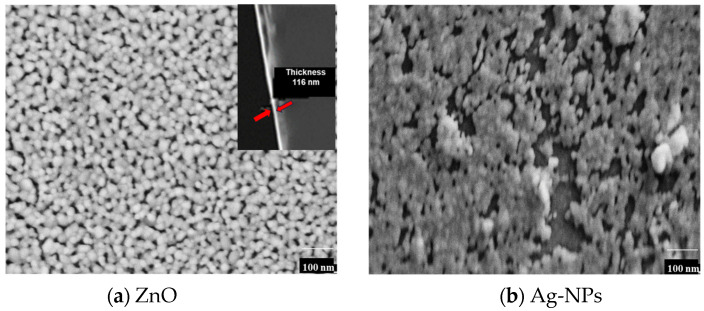
SEM scans of (**a**) microporous ZnO thin film; (**b**) agglomerated Ag-NPS.

The FE-SEM images of a ZnO thin film and silver nanoparticles (Ag-NPs) fabricated by the MPM on silica glass are shown in Figure 2.

**Figure 3:**
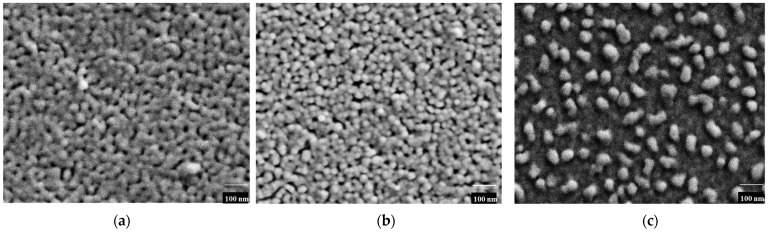
SEM scans of the Ag-NP/ZnO composite thin films at different Ag mol%: (**a**) 10, (**b**) 50, and (**c**) 70, respectively.

- The ZnO surface appears to be microporous and consists of heterogeneous globular grains of different diameters.
- The Ag-NPs on silica glass are agglomerated.
- The film thickness of the ZnO is 116 nm, as confirmed by the inset in Figure 2a.
- The exact thickness of the Ag-NPs film and the composite thin films could not be ascertained due to the uneven deposition of particles, as indicated by the FE-SEM observations (Figure 2b and Figure 3).

Figure 3 shows the surface morphology o thef Ag-NP/ZnO composite thin films as measured by FE-SEM.

- The number of Ag granules (white dots) on the outermost layer of COMP-Ag10 (Figure 3a) was less and more distributed compared to those on the COMP-Ag50 and COMP-Ag70 (Figure 3b,c, respectively).
- Figure 3b depicts the surface morphology of the middle-Ag-level sample, COMP-Ag50, revealing a striking similarity, with the Ag particles uniformly dispersed, exhibiting both spherical and rod-like forms, alongside partially agglomerated Ag-NPs on the thin film.
- Figure 3c depicts a high-Ag-level sample, COMP-Ag 70, characterized by a rough morphology, with globular, rod-like, and significantly agglomerated Ag-NPs on its surface.

#### 3.1.3. Optical Properties

**Figure 4:**
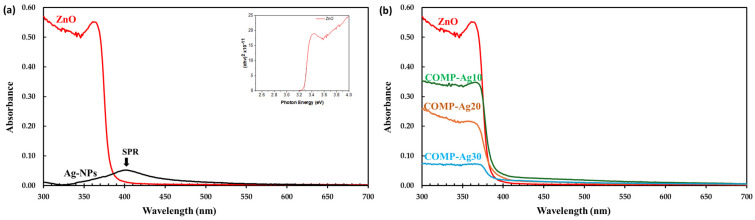
Optical absorption spectra for (**a**) ZnO and Ag-NPs and (**b**) COMP-Ag10–30 thin film fabricated on silica glass.

**Figure 5:**
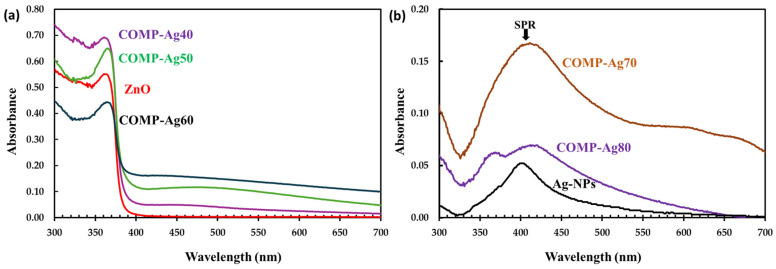
Optical absorption spectra of Ag-NP/ZnO composite thin films at different amounts of Ag mol%: (**a**) 40, 50, and 60; (**b**) 70, 80, and Ag-NPS (100), respectively.

Figure 4a illustrates the UV/Vis absorption spectra for ZnO and Ag-NP, whereas Figure 5b depicts UV/Vis spectra of low-Ag-level COMP-Agn (10 ≤ n ≤ 30) thin films.

- The ZnO thin film had a low-intensity absorption band in the visible spectrum; however, its absorption strength significantly rose in the UV area.
- The direct band gap of ZnO was determined to be 3.4 eV, as illustrated in the inset in Figure 4a.
- A weak and broad absorption peak was seen in the Ag-NP film at a wavelength of around 410 nm, representing the SPR. The characteristic SPR is related to the absorption peak that occurs at 410 nm [26]. The absorption band was similarly detected in COMP-Ag10-30, as presented in Figure 4b; however, the band progressively migrated to longer wavelengths with an increase in Ag concentration.

Figure 5a illustrates the UV/Vis absorption spectra for middle-Ag-level COMP-Agx (40 ≤ x ≤ 60) thin films, whereas Figure 5b depicts UV/Vis spectra of high-Ag-level COMP-Agx (70 ≤ x ≤ 100) thin films.

- The SPR peak near 410 nm is absent in COMP-Ag40 to 60, as shown in Figure 5a; nonetheless, a broad absorption spectrum is observed in the visible range at wavelengths surpassing 410 nm.
- The broad spectral region exhibits a rise in absorption strength with the elevation of the Ag molar concentration in ZnO.
- For composite thin films containing ≥70 mol% Ag, a SPR peak was observed at around 410 nm. Furthermore, a broad absorption spectrum was seen in the visible range, with the intensity of the absorption decreasing as the Ag content increased.
- This broad absorption phenomenon is ascribed to localized surface plasmon resonance (LSPR). Localized surface plasmon resonance (LSPR) transpires when samples of differing dimensions, configurations, and interparticle distances are exposed to light, leading to the creation of significant oscillating electric fields among the metal nanoparticles [15].

### 3.2. Assessment of the Photocatalytic Efficacy of Ag-NP/ZnO Composite Thin Films

**Figure 6:**
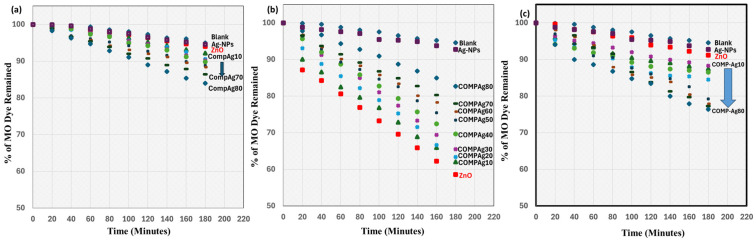
Percentage remaining of the degradation MO solution in the presence of ZnO, various Ag-NP/ZnO composites, and blank upon exposure to (**a**) darkness, (**b**) UV irradiation, and (**c**) visible light irradiation for 180 min.

The photocatalytic efficacy of MO degradation was assessed in a quantitative manner by analyzing the variations in the initial and final absorption spectra intensities of MO, and graphically by calculating the degradation rate of MO (nM min^−1^). The percentages of remaining MO for the degradation of a 1 × 10^−2^ mM (3.27 × 10^−3^ mg/L) aqueous solution of MO using the fabricated photocatalytic thin films and a blank control for 180 min are presented in Figure 6a–c.

- In the dark condition (Figure 6a), the percentages of MO dye that remained for ZnO and Ag-NP exhibit comparable percentages to the blank.
- As a result of this, it was proven that these two samples did not exhibit any catalytic activity when they were exposed to dark conditions.
- The percentages of MO dye that remained for COMP-Ag10, 20, and 30 (low-Ag-level) exhibit minor variations relative to those for COMP-Ag40, 50, and 60 (middle-Ag-level) under the dark condition. However, the percentage change for the remaining MO for COMP-Ag70 and 80 thin films are greater than those observed in low and middle-Ag-level composite thin films.
- The rough surface morphology shown in the SEM images of high-Ag-level composites may facilitate the adsorption of MO into the micropores of these thin films, resulting in a decrease in absorption spectra intensities [36].
- In Figure 6b, the alterations in the percentage of remaining MO diminish as the quantity of Ag in ZnO decreases; specifically, COMP-Ag80 and Ag-NPs (high-level films) exhibit the least photocatalytic activity, whereas ZnO demonstrates the most significant change under UV light irradiation.
- Nearly 62% of the MO remained, i.e., nearly 48% degradation of the MO solution was achieved after 3 h of UV light exposure on ZnO and low-Ag-level films. This resulted from the ample active sites of ZnO in the composite that are photoresponsive to UV irradiation [37].
- The composite thin films containing ≥40 mol% Ag, indicate the regulated alteration between intensities of the MO absorption spectra of the blank and COMP-Ag40 thin films. The increase in Ag concentration in the composites leads to a reduction in the active sites of ZnO, hence diminishing UV responsiveness [24].
- As shown in Figure 6c, the percentage of MO dye that was left behind changed in a more pronounced manner when there was an increase in the amount of Ag present in the ZnO thin films when they were exposed to visible light. ZnO has the lowest photocatalytic efficacy, whereas COMP-Ag70 demonstrates the highest photocatalytic efficacy. The existence of SPR, LSPR, and the surface transformation of the thin films may influence this observed pattern.

The values of the IPCA that were derived from a degradation rate MO solution by photocatalytic effectiveness for each thin film and a blank are provided in Table 1.

- During UV illumination, neither of the samples demonstrate better photoactivity than ZnO thin film; nevertheless, all samples display enhanced photoactivity compared to pure ZnO under visible light irradiation.

**Figure 7:**
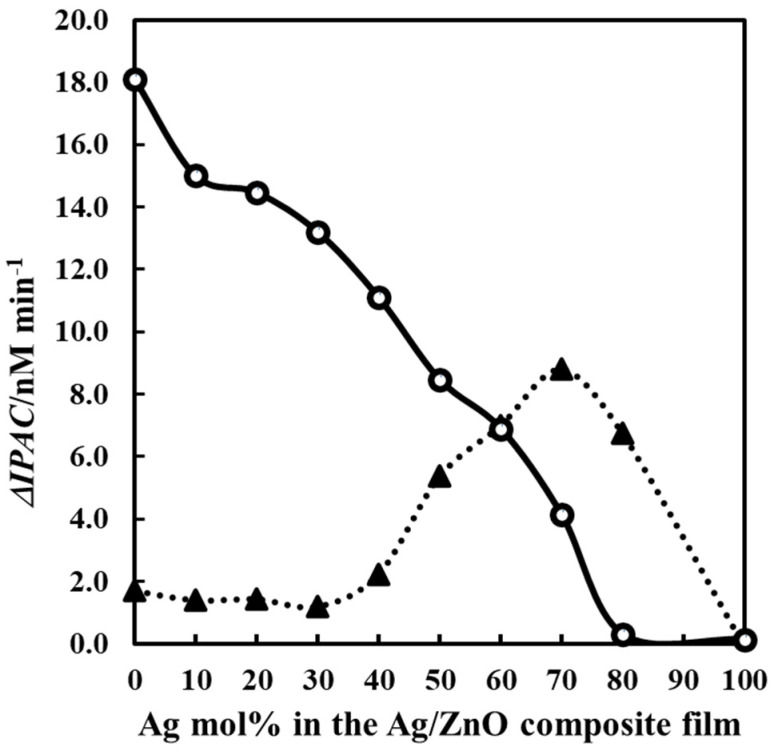
Relationship between ∆IPAC and Ag mol% in the Ag/ZnO composite thin films. The lines are delineated as follows: circle, ∆IPAC under ultraviolet light; triangle ∆IPAC under visible light.

Figure 7 illustrates the relationship between the net photocatalytic action rate (ΔIPCA) of each thin film and the mol% of Ag in the Ag-NP/ZnO composite thin films. The ΔIPCA value for each thin film is determined by subtracting the IPCA obtained under dark conditions from the IPCA recorded during UV- or visible light irradiation.

- The results (∆IPAC) were obtained to evaluate the differences in the IPAC of the degradation of MO during ultraviolet and visible light irradiation vs. those in dark conditions.
- The low-silver-content thin films demonstrate a declining trend in ∆IPCA values when subjected to UV light irradiation, whereas extremely low ∆IPCA values show a slight increase under visible light irradiation as the Ag mol % in the composites rises.
- Composites at the middle-Ag-level demonstrate a consistent reduction in their ∆IPCA values when subjected to UV light irradiation, correlating with an increase in the molar proportion of Ag within the composites.
- In contrast, the same thin films under visible light irradiation demonstrate a noticeable reaction, albeit slightly less than the reduced UV responsiveness of the equivalent thin film samples.
- A local maximum in ∆IPAC is observed with elevated silver concentrations for visible light sensitivity.
- A sudden decrease in the ∆IPCA value under both ultraviolet and visible light irradiation is apparent between 80 and 100 mol% of silver concentration.

## 4. Discussion

### 4.1. Chemical, Morphological, and Optical Characterization of Fabricated Thin Films

The fabrication of Ag-NPs/ZnO composite thin films is conducted using many procedures, each presenting distinct advantages and disadvantages [38]; it is still uncertain which will ultimately be the most effective [27]. The remarkable stability of the Zn-EDTA precursor solution employed in this study, which can last for a year, provides significant practical benefits to the MPM compared to sol–gel [6]. The MPM has recently been used to synthesize precursor solutions with superior miscibility, as evidenced in several precursor solutions, such as Cu-EDTA [39] and Ti-EDTA [28], during the fabrication of metal oxide thin films.

The XRD patterns (Figure 1) validate the presence of metallic silver and hexagonal wurtzite structure in the fabricated composites, indicating an increase in the intensity of the metallic silver peaks while the hexagonal wurtzite peak diminishes with the increasing mol% of silver in the ZnO matrix [6,40]. The incorporation of Ag did not alter the XRD peak positions of wurtzite ZnO, indicating no expansion in the lattice parameter. The amount of Ag-NPS on the surface of COMP-Ag10 was lower and more distributed compared to COMP-Ag50 and COMP-Ag70. COMP-Ag50 displays uniformly scattered Ag-NPs, characterized by both spherical and rod-like shapes, as well as being partially agglomerated on the surface of the thin film. COMP-Ag 70, distinguished by a coarse surface morphology and markedly agglomerated silver particles on its surface. The utilization of nanosized silver particles in lieu of micro-sized particles is crucial for integrating Ag-NPs into a semiconductor matrix, as nanoparticles potentially mitigate the settling issue observed in certain micro-sized Ag particle systems, which can hinder homogeneous distribution and lead to the agglomeration of Ag nanoparticles [41].

The band gap of ZnO was determined to be 3.4 eV, as illustrated in the inset in Figure 4a. The band gaps of the composite thin films closely resemble those of ZnO, since the absorption band edge remained unchanged with the incorporation of silver in the ZnO matrix (Figure 4b and Figure 5a) [42]. This indicates that Ag-NPs did not increase or decrease the band gap of ZnO in the composites. COMP-Ag10–30 exhibit a weak or undetectable surface plasmon resonance (SPR) peak at approximately 400 nm (Figure 4b), whereas the Ag NP film displays this peak at 410 nm (Figure 4a). When an Ag-NP is treated with visible light, a substantial oscillating electric field is detected around the metal particles [21,31,32]. The absorption band in this region corresponds to the distinctive SPR of Ag-NPs. The COMP-Ag40–60 thin films exhibit pronounced and broad absorption attributed to SPR in the visible light spectrum. The extensive absorption by the COMP-Ag40–60 thin films escalates with a rise in Ag concentration. Numerous studies indicate that broad absorption is associated with a tip-to-tip plasmon mode and intraparticle plasmonic coupling of tip and cavity resonances, as well as the scattering of incident light [15,17,30]. The COMP-Ag70–80 thin films exhibit two overlapping SPR peaks at around 394 nm, accompanied by significant absorptions throughout the broad visible light spectrum. The extensive absorption shown by the COMP-Ag60 thin films diminished as the Ag concentration increased to 70 and 80. This observation is attributable to localized surface plasmon resonance (LSPR). LSPR occurs when samples of varying size, shape, and separation distance are illuminated by light, resulting in the generation of substantial oscillating electric fields between the metal nanoparticles [15]. SEM (Figure 2) micrographs confirm that the composite exhibits Ag NPs of varying sizes and shapes.

Based on the observed morphological and optical properties, the fabricated composite thin films may be classified into the following three categories according to the silver content: low-Ag-level (COMP-Ag10–30 (10 ≤ Ag mol% ≤ 30)), middle-Ag-level (COMP-Ag40–60 (40 < Ag mol% < 60)), and high-Ag-Level (COMP-Ag70–80 (70 ≤ Ag mol%)) thin films. These three categories will be utilized to discuss the photocatalytic efficacy of the thin films.

### 4.2. Photocatalytic Efficacy of Ag-NP/ZnO Thin Films Under Ultraviolet Light Exposure

Figure 7 illustrates that the ΔIPCA of the composites diminished with the increasing mol% of Ag in the ZnO matrix under UV irradiation. ZnO has the highest ΔIPCA values. This is expected, since its band gap was determined to be 3.4 eV, and it is also attributed to the hexagonal wurtzite structure of ZnO identified in the thin films via XRD [6]. The sharp decline in photocatalytic efficacy transpires between 10 and 80 mol% of the Ag content. The observed decline in photocatalytic activity is attributable to the reduction in hexagonal wurtzite structure of ZnO content in the composites as the Ag content increases [24]. This trend should be enhanced by augmenting the Ag content in the composites, as the deposition of Ag particles on the ZnO surfaces occurs markedly, even with a homogenous distribution of Ag nanoparticles [26]. The incorporation of Ag-NPs into the ZnO matrix did not affect the band gap of ZnO, as evidenced by the absorption spectra. The decline in photocatalytic activity of the composites under UV irradiation is directly related to the increasing amount of Ag nanoparticles that obscure the active sites of ZnO [24]. COMP-Ag80 has lowest photocatalytic activity under ultraviolet. The ZnO active site seems to be entirely obscured by the agglomerated Ag-NPs, resembling the structure of pure Ag-NPs; hence, no photocatalytic activity is seen.

### 4.3. Photocatalytic Efficacy of Plasmonic Ag-NP/ZnO Composite Thin Films Under Visible Light Exposure

As illustrated in Figure 7, the ΔIPCA of the composites under visible light irradiation indicates that the low-Ag-level samples, specifically COMP-Ag10, COMP-Ag20, and COMP-Ag30, exhibit values nearly identical to those of pure ZnO. Furthermore, there are two points of inflection. One manifests at 30 mol% as a local minimum, while another occurs at 70 mol% as a local maximum. This is within the range of the middle-Ag-level category. The enhancement in visible light sensitivity of the composites from 30% to 70% was not only significant but also considerable in scale; the overall photocatalytic activity of the composites under visible light irradiation exhibits a single point of inflection based on the amount of Ag present in the composites. An increase in the silver content in composite thin films is known to enhance absorption intensity [26]. The lamp utilized to investigate the degradation of MO aqueous solution in this investigation emits 5.9 mWcm^−2^ across a broad spectrum of 400 nm to 700 nm, which is adequate to activate visible light photocatalytic activity. All the middle-Ag-level composites demonstrate a broad range of SPR absorption, indicating their ability to respond to visible light. Thus, their photocatalysis activity was observed to increase with Ag content due to the plasmonic properties of the composites. Although the overall photocatalytic activity improved with the increasing Ag content in the composite, a significant decline in the ΔIPCA value is seen between 70 and 80% (high-Ag-level range). In high-Ag-level composites, the photocatalytic activity exhibits a local maximum for COMP-Ag70 due to the SPR peak at 410 nm, which subsequently diminishes for COMP-Ag80 as the SPR peak likewise declines.

### 4.4. Proposed Mechanism for the Photocatalytic Capabilities of Plasmonic Ag-NP/ZnO Composite Thin Films

When the composite contains 70 Ag mol% or less, the reported SPR and LSPR absorptions can be discerned. The precise SPR peak was observed at around 410 nm, attributable to the inherent optical resonance of spherical Ag-NPs. While this peak is concentrated at 410 nm, approximately one-third of its area in these composite thin films falls within the visible light spectrum (≥400 nm). Conversely, other composites exhibit a broad absorption spectrum attributable to localized surface plasmon resonance (LSPR). Thus, this indicates their ability to respond to visible light. Therefore, the charge transfer mechanisms that occurred in the plasmonic Ag-NP/ZnO thin films under visible light conditions are summarized as follows [42]:

(i) Electrons from Ag nanoparticles gain access to higher energy levels in the band due to localized surface plasmon resonance (LSPR).
(ii) These electrons are introduced into the conduction band of ZnO.
(iii) Electrons migrate to catalyzing active regions at the semiconductor/ZnO interface, facilitating chemical reactions that will result in the oxidation of MO (22).

Typically, Ag/metal oxide thin films exhibit limited longevity due to the susceptibility of Ag-NP on ZnO to the oxidation process when exposed to visible light irradiation; therefore, plasmonic photocatalytic activity is not detected unless the Ag core is encased in a silica (SiO_2_) shell [43] or shielded with an Al_2_O_3_ nano mask [44] to inhibit the oxidation process from direct interaction with ZnO. The MPM facilitates plasmonic photocatalysis while leaving the metallic Ag core in the composite unexposed.

## 5. Conclusions

This work examines the photocatalytic effectiveness of silver nanoparticle-doped zinc oxide (Ag-NP/ZnO) composite thin films (COMP-Agn) in degrading methyl orange (MO) under ultraviolet and visible light exposure. The composite films, produced via the molecular precursor technique (MPM) with different silver contents (0–80 mol%), were spin-coated onto silica glass substrates at 600 °C. X-ray diffraction (XRD) examination verified the emergence of hexagonal wurtzite ZnO crystallites in conjunction with cubic silver crystals. The strength of the ZnO diffraction peak diminished as the Ag content increased. Scanning electron microscopy (SEM) demonstrated the efficient incorporation of Ag nanoparticles into the ZnO matrix, exhibiting diverse morphologies ranging from spherical to rod-like, contingent upon the silver concentration. UV/Vis spectroscopy revealed a surface plasmon resonance (SPR) peak at around 410 nm for the Ag-NP/ZnO films, which increased visible light absorption. The photocatalytic activity was assessed by observing the degradation of MO under ultraviolet and visible light. Under UV illumination, pure ZnO demonstrated the greatest photocatalytic efficacy, which diminished with increasing silver concentration due to the obstruction of active ZnO sites by Ag-NP agglomeration. Conversely, when exposed to visible light, composite films containing moderate silver concentrations (40–60 mol%) exhibited improved photocatalytic efficacy, ascribed to surface plasmon resonance-induced electron transport from Ag nanoparticles to the ZnO conduction band. The research classifies the composite films into three categories according to silver content, as follows: low-Ag (10–30 mol%), mid-Ag (40–60 mol%), and high-Ag (70–80 mol%). The mid-Ag-level films demonstrated the most substantial enhancement in visible light photocatalysis. The hypothesized method entails electron excitation via surface plasmon resonance (SPR), followed by electron transfer to the conduction band of ZnO, which facilitates the decomposition of methyl orange (MO). This study emphasizes the capability of Ag-NP/ZnO composite thin films to improve photocatalysis under visible light and elucidates the correlation between silver concentration, structural characteristics, and photocatalytic efficacy. The MPM approach has shown benefits in precursor stability and the effective incorporation of Ag nanoparticles into the ZnO matrix, presenting a promising pathway for improved photocatalytic materials. Nonetheless, numerous obstacles persist. These include having to adjust the dispersion of Ag nanoparticles to avert agglomeration at elevated concentrations, which may impede UV photocatalytic activity. Furthermore, the long-term durability and reusability of the composite films under operational settings require additional examination.

## Figures and Tables

**Table 1:** The index of photocatalytic action rate (IPAC) of the rate of degradation of the 0.01 nM MO solution for each thin film and a blank.

Notation		IPAC in nM min^−1^	
	Visible ^1^	UV ^1^	Dark ^1^
**Control**	3.1 (0.3)	3.3 (1.3)	2.9 (0.4)
**Pure ZnO**	5.1 (0.6)	21.5 (5.3)	3.4 (0.3)
**COMP-Ag10**	5.9 (0.3)	19.5 (3.9)	4.5 (0.3)
**COMP-Ag20**	6.3 (0.7)	19.3 (2.2)	4.9 (0.3)
**COMP-Ag30**	6.9 (0.8)	18.9 (1.5)	5.7 (0.1)
**COMP-Ag40**	8.1 (0.8)	16.9 (1.9)	5.8 (0.2)
**COMP-Ag50**	11.4 (2.7)	14.5 (3.2)	6.0 (0.7)
**COMP-Ag60**	13.4 (2.0)	13.3 (2.9)	6.4 (0.5)
**COMP-Ag70**	16.5 (2.9)	11.8 (2.5)	7.7 (1.8)
**COMP-80**	15.9 (2.9)	9.5 (0.4)	9.2 (0.8)
**Ag-NPs**	3.4 (0.5)	3.6 (0.3)	3.5 (0.3)

^1^ The standard deviations are enclosed in brackets.

## Data Availability

Data are contained within the article.

## Abbreviations

The following abbreviations are used in this manuscript:

MPM	Molecular precursor method
MO	Methyl orange
COMP-Ag10	Silver/ZnO composite thin film with 10 mol% of silver
Ag-NPs	Silver nanoparticles
SPR	Surface plasmon resonance
LSPR	Localized surface plasmon resonance
IPCA	Index of photocatalytic action rate
ΔIPCA	Net index of photocatalytic action rate
